# The promotion and suppression of DNA charge neutralization by the cosolute ectoine

**DOI:** 10.1039/c9ra09355a

**Published:** 2019-12-12

**Authors:** Benteng Chen, Yanwei Wang, Guangcan Yang

**Affiliations:** Department of Physics, Wenzhou University Wenzhou 325035 China wangyw@wzu.edu.cn yanggc@wzu.edu.cn +86-577-86689010 +86-577-86689033

## Abstract

Ectoine, a cosolute and osmolyte, is used by extremophilic microorganisms to maintain an osmotic equilibrium of cells with their surrounding medium under conditions of extreme salinity or thermal and pressure stresses. It is also considered a protectant of biomolecules such as protein and DNA in cells. In the present study, we investigate its influence on DNA charge neutralization and compaction through dynamic light scattering (DLS), atomic force microscopy (AFM) and single molecular magnetic tweezers (MT). We found that ectoine can promote DNA charge neutralization induced by multivalent cations at mild cosolute concentration in solution. When the concentration of ectoine is high enough, however, a mixed effect of promotion and suppression can be found under the same ionic conditions. In this case, the electrophoretic mobility (EM) of DNA is promoted in the region of low cation concentration, while suppressed in the region of high counterionic concentration. The charge neutralization of DNA by ectoine is also related to DNA compaction. The promotion and suppression of DNA compaction by ectoine was observed by AFM imaging. The condensed structure of DNA becomes more compact and then loose once more with the increasing concentration of ectoine. Meanwhile, the condensing forces of DNA measured by magnetic tweezers shows the same trend as does the DNA EM. We explained the experimental findings through the combined effect of two intrinsic features of ectoine, preferential exclusion and enhancement of the dielectric constant of the medium.

## Abstract 2

Ectoine can promote DNA charge neutralization at mild cosolute concentration in solution. When the concentration of ectoine is high enough, however, a mixing effect of promotion and suppression can be found in the same ionic condition.

## Graphical abstract

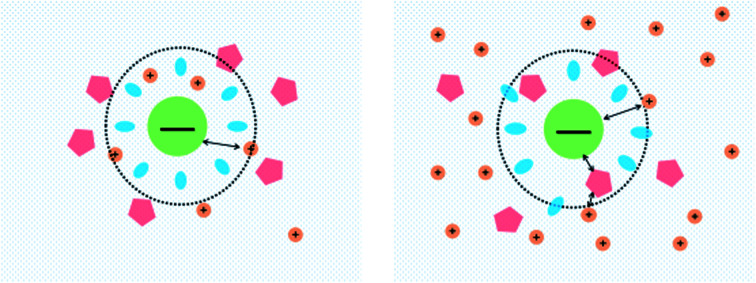

## 1. Introduction

**Fig. 1:**
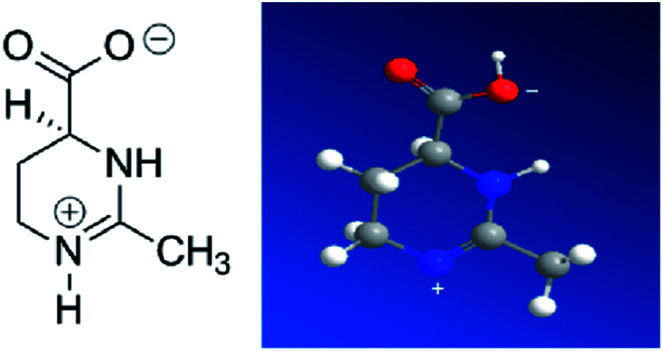
Molecular structure of ectoine.

Ectoine is initially produced and accumulated in high concentrations by halophilic and halotolerant bacteria to maintain the equilibrium of cytoplasm with the surrounding medium under extreme condition such as salinity, thermal and pressure stress.^1–3^ This is achieved by maintaining the balance of the chemical potential between the cytoplasm inside the cell and its surrounding through ectoine, instead of adjusting salt concentrations.^3^ It is also widely used for pharmaceutical and cosmetic products due to its strong water-binding behaviour.^4–7^ For example, ectoine has been tested to be effective in protecting the skin against water loss and desiccation.^8^ In the test, the skin pre-treated by ectoine becomes less susceptible to damage by detergents and subsequent water loss. Ectoine is not only a protectant of biomolecules in extreme environment, like freezing, drying or high temperature,^2^ but also a compatible solute^9^ (or cosolute) as it does not negatively affect the cell metabolism. Ectoine is a small organic molecule with neutral charge and low toxicity even at high concentrations. The molecular mass of ectoine is 142.2, and its structure is shown in Fig. 1. Recent experimental and simulation investigations have shown that ectoine can enhance pH-dependent structural changes of DNA *in vitro* and has a significant influence on water dielectric property for regulating the interaction between protein and DNA.^10–14^ The temperature and pressure dependent conformational dynamics of nucleic acids is also modified in the presence of crowders and osmolytes.^15^ It has been experimentally shown that ectoine lowers the melting temperature of double-stranded DNA and increases the thermal stability of DNA polymerases at elevated temperatures.^16^ DNA is not only a biomolecule carrying genetic information but also a highly charged biological polyelectrolyte in solution. The highly charged and stiff polymer can be compacted *in vivo* and *in vitro* by many counterions, some proteins and many other condensing agents.^17–19^ Understanding the interaction between DNA and these agents is not only important for investigating fundamental biological processes such as chromatin and chromosome assembling, but also useful for developing new gene vehicles in therapeutic applications.^20–22^ The DNA compaction is closely related to its charge neutralization since Coulombic repulsion is quite strong if the charges of DNA segments are not compensated or screened.^23–25^ In our previous work, we found that many factors including the types of counterions, pH in solution and hydrophobicity of agents have significant influence on DNA compaction and its charge compensation.^26–31^ In some cases, over compensation or charge inversion occurs when the charge of counterions surrounding the DNA skeleton is more than the polyelectrolyte itself.^32–35^ Up to now, the interaction mechanism between cosolutes and DNA, the role of cosolutes in DNA compaction and charge neutralization, have been less explored, and thus, are the main topics of the current work.

In the present study, we use dynamic light scattering (DLS), atomic force microscopy (AFM) and single molecular magnetic tweezers (MT) to investigate the influence of ectoine on the charge neutralization and compaction of DNA systematically. As a compatible solute, ectoine plays the role of protector of biomolecules since it can enhance the stability of proteins, membranes and other biomolecules.^2–4^ The underlying mechanisms for the strong protective properties of ectoine is assumed to be dependent on its hydration exclusion. On the other hand, ectoine is a zwitterionic electrolyte, which increases the static relative permittivity of the medium, lowering the effective screening of the long-range Coulombic interactions among ions in ectoine-containing solutions.^36^ Our previous study shows that zwitterions in solution hinders the charge neutralization of DNA by cations in solution.^26^ Thus, we believe that the interplay among many effects of ectoine on DNA leads to certain new and interesting behaviours of DNA charge neutralization and compaction.

This paper is organized as follows: in Section 2, we present the results and discussions on EM of DNA in solution of counterions by DLS, DNA morphologies by AFM, and tethering forces by MT. The materials, methods and procedures are presented in Section 3. Finally, some conclusions are drawn in Section 4.

## 2. Results and discussions

### Electrophoretic mobility of DNA in solution with ectoine

To investigate the charge neutralization or compensation of DNA, we measured the EM of DNA in solution at various cationic and ectoine concentrations by DLS. The mobility reflects DNA surface charge, including its bare charge and the charge of the corresponding condensed counterions. Charge inversion occurs when the mobility switches its sign, implying the condensed charge is more than the bare charge of DNA. In the mobility measurement by DLS, an electric field is switched on and off periodically and applied to the solution, the phase of laser light scattered from the DNA condensates is recorded over time. When the charged DNA condensates drift in the electric field yield, the phase of scattered light evolves at a rate proportional to their EM. This velocity is measured using a laser interferometric technique called phase analysis light scattering (PALS). This enables us to infer the EM of DNA. This approach is more efficient and convenient than the usual gel electrophoresis. Specifically, when an electric field is applied to DNA solution, the negatively charged DNA molecules migrate towards positive electrode, and the counterions move in an opposite direction. The polarity of the applied electric field is periodically reversed to eliminate electroosmotic flows, as shown in ref. 37. The EM *μ* of DNA is given by.^38^1
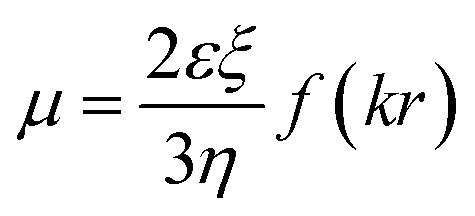
where *ξ* is the zeta potential, *ε* is the dielectric constant of the solvent, *η* is the viscosity coefficient of the solvent, and *f*(*kr*) is the Henry function, where *r* is the particle radius, and 1/*k* is Debye length. Thus, we can also obtain the zeta potential of DNA condensates from the measured EM by eqn (1) if the viscosity of solution is known.

**Fig. 2:**
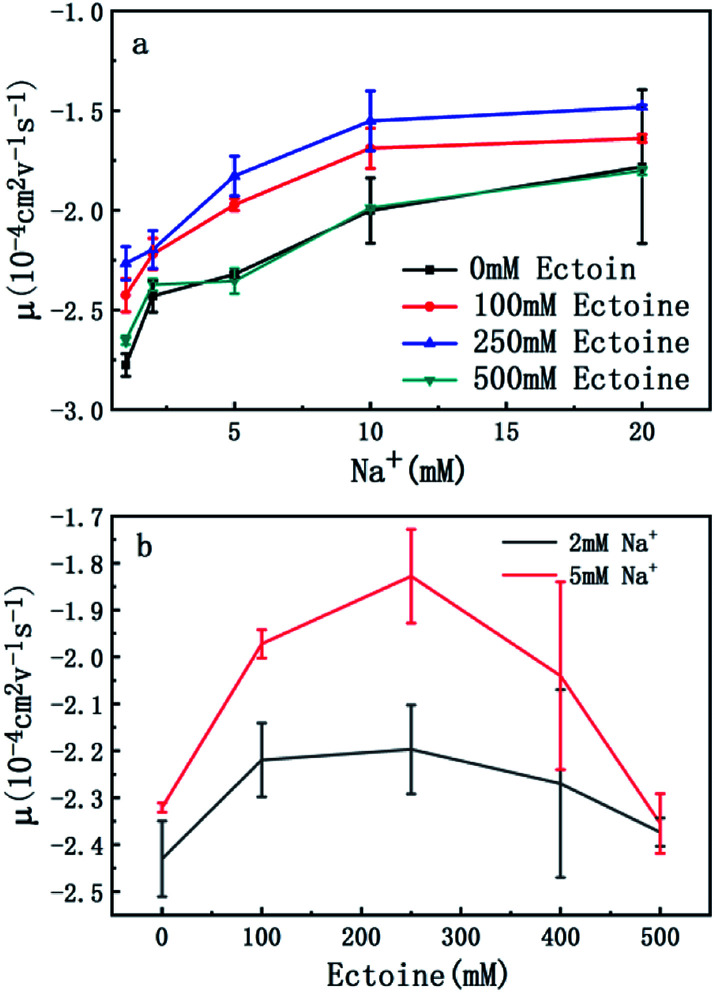
(a) EM of DNA *versus* the concentration of Na^+^ with 0 mM, 100 mM, 250 mM and 500 mM ectoine respectively. (b) EM of DNA *versus* the concentration of ectoine with 2 mM and 5 mM Na^+^ respectively in solution. 10 mM Tris (pH = 7.5) buffer is used, and the concentration of DNA is 1 ng μL^−1^. The error bars represent the corrected sample standard deviation.

The measured electrokinetic properties of DNA as a function of counterion concentration in solution are shown in Fig. 2 at various ectoine concentrations. In Fig. 2(a), the EM (EM) of DNA is plotted *versus* the concentration of sodium ions. We can see that the EM of DNA complex increases monotonously with the concentration of sodium ions in solution, as expected. For example, the mobility increases gradually from −2.7 × 10^−4^ cm^2^ V^−1^ s^−1^ (10^−4^ cm^2^ V^−1^ s^−1^ is the unit of the EM and is used consistently below thus will be omitted for clarity) to −1.7, when the concentration of sodium ions goes from 1 mM to 20 mM if no ectoine is added in solution. When 100 mM ectoine is introduced into the solution, the changing tendency of EM of DNA remains same as without ectoine, but the mobility curve is entirely shifted upwards. For example, the mobility of DNA in solution of 100 mM ectoine is promoted to −1.9 at the sodium ionic concentration 5 mM from −2.3 in absence of ectoine. When we increase the concentration of ectoine further to 250 mM, the promotion effect still exists but becomes less efficient. For example, when we fix the concentration of Na^+^ at 10 mM and 100 mM ectoine is added to the solution, the EM of DNA goes up from −2.0 to −1.6, and the corresponding promotion is about 0.40. In the same ionic condition, the mobility is further promoted from −1.6 to −1.5 when the concentration of ectoine is increased from 100 mM to 250 mM. The corresponding promotion value is only 0.10, significantly less than the former value. When we add more ectoine to solution so that its concentration reaches 500 mM, we can see that the EM of DNA almost recovered to the original value, before the introduction of the cosolute, implying the promotion is almost disappeared. This effect can be seen more clearly in Fig. 2(b), where the mobility is plotted *versus* ectoine concentration with a fixed cationic concentration. We can see that the mobility of DNA goes up initially, reaches its maximum, then retreats to about the original value when we fixed the concentration of Na^+^ at 2 mM and altered the concentration of ectoine from 0 to 500 mM. If we increased the concentration of Na^+^ to 5 mM, a similar phenomenon can be observed. In other words, ectoine at high concentration has a suppression effect on the charge neutralization of DNA in counterionic solution.

**Fig. 3:**
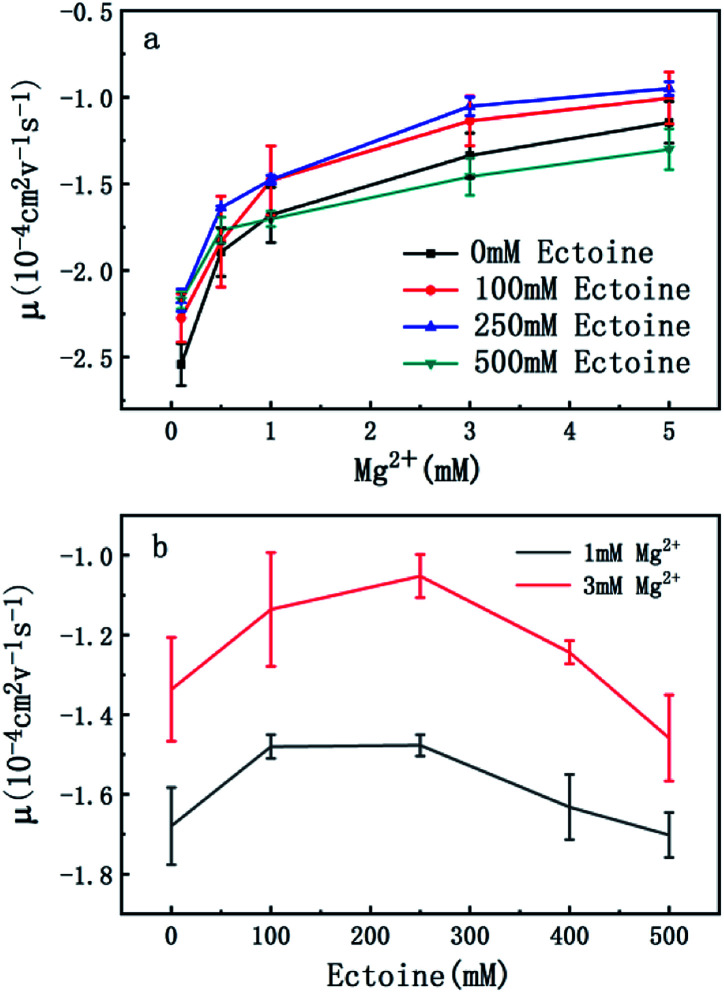
(a) EM of DNA *versus* the concentration of Mg^2+^ with 0 mM, 100 mM, 250 mM and 500 mM ectoine respectively. (b) EM of DNA *versus* the concentration of ectoine with 1 mM and 3 mM Mg^2+^ respectively in solution. The error bars represent the corrected sample standard deviation.

In order to explore the promotion of charge neutralization of DNA further, we measured the EM of DNA in solutions of divalent counterion with and without ectoine. The mobility of DNA is plotted *versus* the concentration of Mg^2+^, shown in Fig. 3(a). Similar to the case of monovalent ion, we can see the EM of DNA complex increases monotonously with the increasing concentration of Mg^2+^ ions in solution. For example, the EM increases gradually from −2.5 to −1.1 when the concentration of Mg^2+^ ions goes from 0.1 mM to 5 mM if no ectoine is added in solution. When 100 mM ectoine is introduced into the solution, the changing tendency of EM of DNA is the same as the case of absence of ectoine, but the mobility curve is again entirely shifted upward. For example, the mobility of DNA in solution of 100 mM ectoine is promoted to −2.2 at the Mg^2+^ ions concentration 0.1 mM from −2.5 in absence of ectoine. When we increase the concentration of ectoine further to 250 mM, the promotion effect of charge neutralization of DNA is still effective but becomes weaker. When we fix the concentration of Mg^2+^ to 3 mM and 100 mM ectoine is added to the solution, the EM of DNA goes up from −1.3 to −1.1, implying the promoting value is about 0.20. In the same ionic condition, the mobility is further promoted from −1.1 to −1.0 when the concentration of ectoine is increased from 100 mM to 250 mM. The corresponding promoting value is only 0.10, significantly less than the former value 0.20. When we add more ectoine into solution (500 mM), we see a different scenario. In the range of low concentration of Mg^2+^ (<1 mM), the EM of DNA is still promoted in presence of ectoine, but the amplitude of promotion decreases gradually and approaches zero at [Mg^2+^] = 1 mM with the increasing concentration of divalent counterion. Crossing the critical point, when we increase the concentration of Mg^2+^ further, the mobility of DNA becomes lower than its corresponding value in the absence of ectoine, implying charge neutralization of DNA is suppressed. This effect can be seen more clearly in Fig. 2(b), where the mobility of DNA goes up initially to its maximum, then approaches the original value when we fixed the concentration of Mg^2+^ at 1 mM and 3 mM respectively while increasing concentration of ectoine from 0 to 500 mM.

**Fig. 4:**
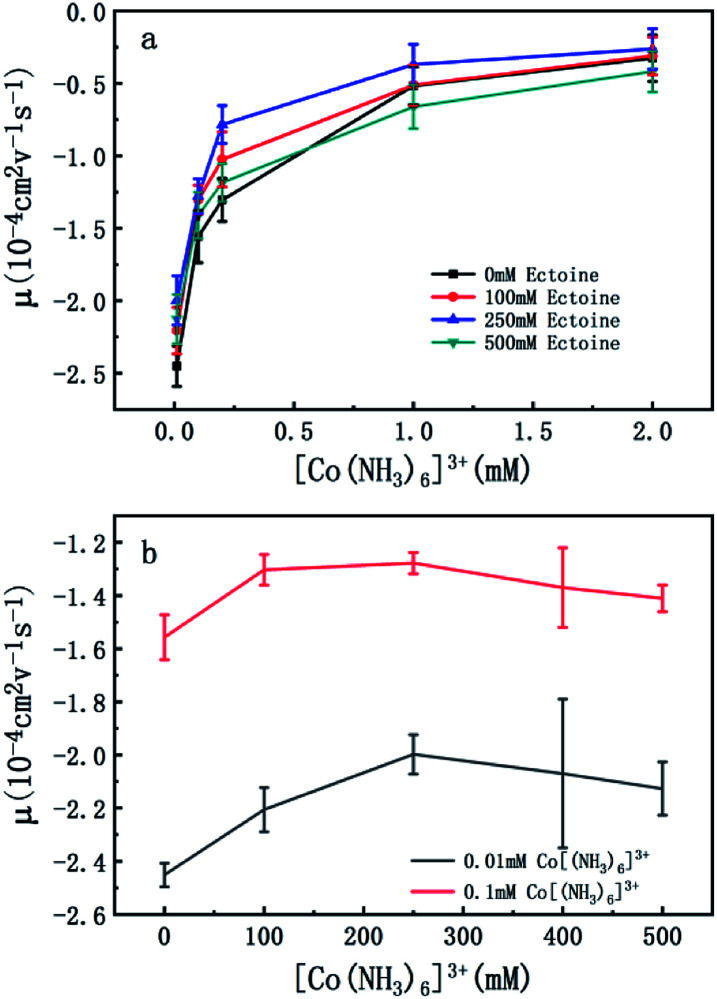
(a) EM of DNA *versus* the concentration of [Co(NH_3_)_6_]^3+^ with 0 mM, 100 mM, 250 mM and 500 mM ectoine respectively. (b) EM of DNA *versus* the concentration of ectoine with 0.01 mM and 0.1 mM [Co(NH_3_)_6_]^3+^ respectively in solution. The error bars represent the corrected sample standard deviation.

To confirm the universality of the promotion and suppression of charge neutralization of DNA by the compatible solute ectoine, we further investigated the case of trivalent counterion. The EM of DNA is plotted *versus* the concentration of [Co(NH_3_)_6_]^3+^ ions in Fig. 4(a) and (b). With the increasing concentration of [Co(NH_3_)_6_]^3+^ ions in solutions, we can see the EM of the DNA complex increases monotonously. For example, the EM increases gradually from −2.4 to −0.32 when the concentration of [Co(NH_3_)_6_]^3+^ ions goes from 0.01 mM to 2 mM if no ectoine is added in solution. When 100 mM ectoine is introduced into the solution, the changing tendency of EM of DNA is the same as the case of absence of ectoine, but the mobility curve is entirely shifted upwards. For example, the mobility of DNA in solution of 100 mM ectoine is promoted to −1.0 at the [Co(NH_3_)_6_]^3+^ ions concentration 0.2 mM from −1.3 in absence of ectoine. When we increase the concentration of ectoine further to 250 mM, the promotion effect of charge neutralization of DNA is still effective but becomes weaker. For example, when we fix the concentration of [Co(NH_3_)_6_]^3+^ to 0.1 mM and 100 mM ectoine is added to the solution, the EM of DNA goes up from −1.5 to −1.3, implying the promoting value is about 0.2. In the same ionic condition, the mobility is further promoted from −1.3 to −1.2 when the concentration of ectoine is increased from 100 mM to 250 mM. The corresponding promoting value is only 0.1, significantly less than the former value 0.2. When we add more ectoine to solution further so that its concentration reaches 500 mM, we can see that the EM of DNA almost recovered to the original value in absence of the cosolute, implying the promotion is almost disappeared. Once again, as we can see in Fig. 4(b), where the mobility of DNA climbs up initially, reaches the maximum, and goes down to about the original value when we fixed the concentration of [Co(NH_3_)_6_]^3+^ to 0.01 mM and 0.1 mM while varying concentration of ectoine from 0 to 500 mM. It is remarkable that all the DNA, in the range of ionic concentrations of the present study, is in the free draining regime even in buffer of a few mM of Na^+^ ions, where the EM is only proportional to the charge density after Manning condensation.^39^ Of course, viscosity is also a factor affecting the EM since they are related by eqn (1). For the buffer of 2 mM [Co(NH_3_)_6_]^3+^ with ectoine (0 mM, 100 mM, 250 mM, 500 mM), the measured viscosities are 0.82, 0.84, 0.85 and 0.86 cP respectively. The corresponding EM of DNA in the same buffer are −0.32, −0.31, −0.26 and −0.42 respectively. We can see that the viscosity increases slightly with increasing concentration of ectoine. Nevertheless, the value of viscosity increases less than 5% even at the highest concentration accessible experimentally, corresponding to the decrease of the mobility by the same percentage. Therefore, dependence of the charge neutralization of DNA on the concentration of ectoine is not affected much by the slight increase of the viscosity of solution.

**Fig. 5:**
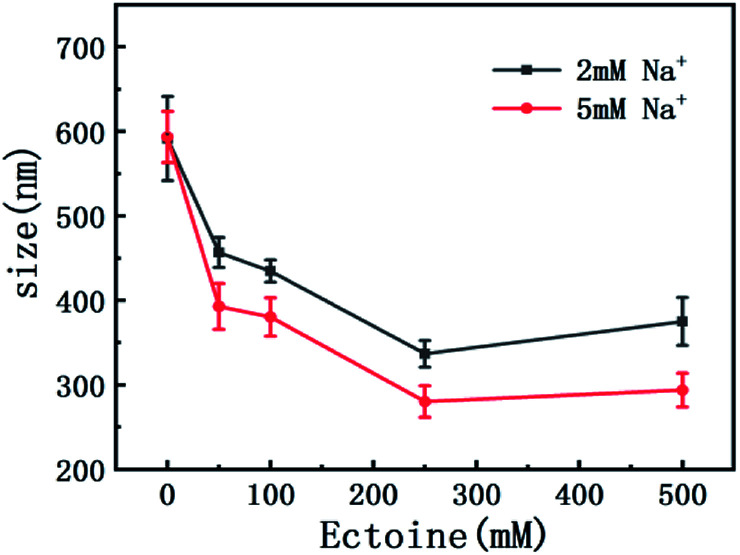
The size of DNA at different concentrations of Na^+^ (2 mM, 5 mM) as a function of ectoine concentration. The error bars represent the corrected sample standard deviation.

**Fig. 6:**
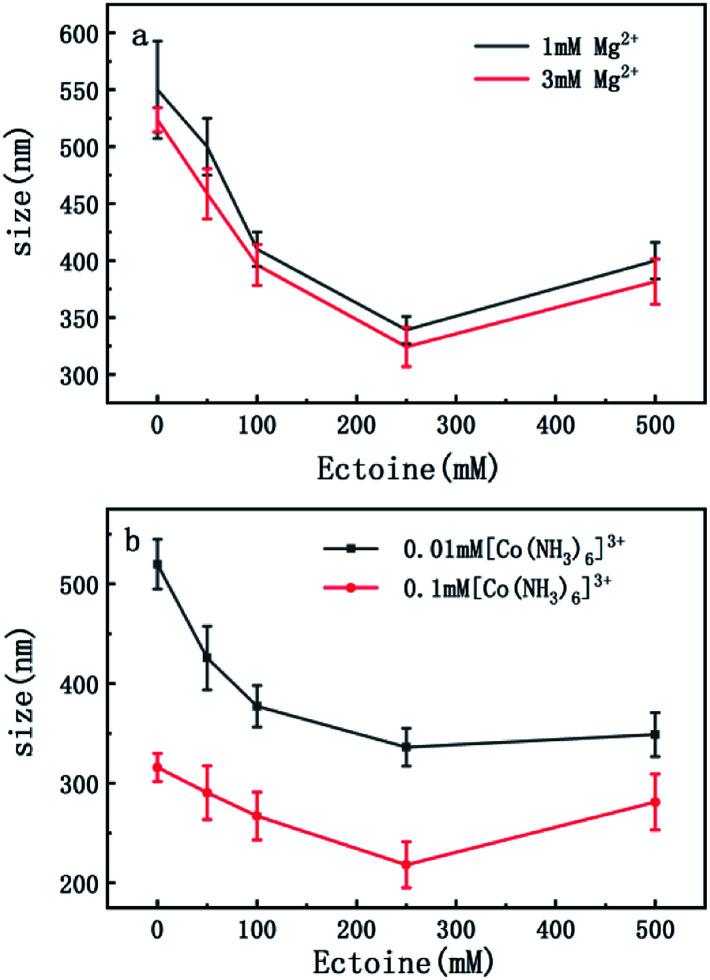
(a) The size of DNA at different concentrations of Mg^2+^ (1 mM, 3 mM) as a function of ectoine concentration. (b) The size of DNA at different concentrations of [Co(NH_3_)_6_]^3+^ (0.01 mM, 0.1 mM) as a function of ectoine concentration. The error bars represent the corrected sample standard deviation.

On the other hand, charge neutralization of DNA is closely related with its compaction since Coulombic repulsion between the charged segments of DNA play an important role in DNA compaction or condensation. Usually, more DNA charge is compensated or neutralized by counterion in solution corresponds to more compact of DNA conformation. Thus, we measured the particle sizes of DNA under similar conditions of counterion and compatible cosolutes. The results are shown in Fig. 5 and 6, corresponding to the cases of monovalent, divalent and trivalent counterions respectively. In Fig. 5, we can see that the size of DNA decreases and approaches an almost constant value with the increasing ectoine concentration under the condition of fixed monovalent cations. The DNA sizes changes slightly in the cases of di- and trivalent counterions, as shown in Fig. 6, where the sizes of DNA increase in the high concentration range of ectoine. This result is consistent to the measurement of EM of DNA presented in the last paragraphs.

### The morphologies of DNA by atomic force microscopy

**Fig. 7:**
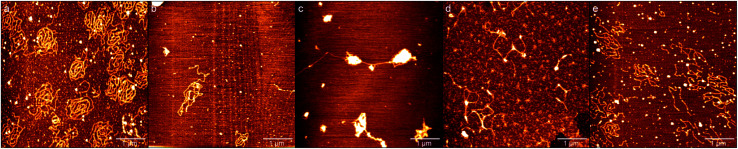
AFM images of DNA at different concentrations of ectoine and a fixed 0.01 mM [Co(NH_3_)_6_]^3+^. (a) Without ectoine. (b) 50 mM Ectoine. (c) 200 mM Ectoine. (d) 300 mM Ectoine. (e) 500 mM Ectoine. Buffer solution of Tris (10 mM, pH = 7.5) was used for all measurement.

In order to investigate the influence of ectoine on the conformation of DNA, we used AFM to observe the morphology of DNA at various concentrations of ectoine. [Co(NH_3_)_6_]^3+^ is a typical trivalent counterion that compacts DNA quite easily. Its critical concentration for condensing DNA is about 0.02 mM.^31,40^ Thus, we imaged DNA morphologies by fixing the concentration of [Co(NH_3_)_6_]^3+^ to 0.01 mM, much lower than the critical concentration, but adjusting the ectoine concentration as a controlling parameter. The images are shown in Fig. 7. In Fig. 7(a) where the concentration of [Co(NH_3_)_6_]^3+^ (10 mM Tris, pH = 7.5) is 0.01 mM in absence of ectoine, we can see the naturally extended DNA on the fresh mica surface but with slightly shrinking. When 50 mM ectoine is added into the DNA solution, as shown in Fig. 7(b), we can see that the shrinking of DNA becomes more apparent compared with the case without ectoine. If we continue to increase the concentration of ectoine, some condensed cores form while some DNA around the condensed cores are still in the coiled conformation, as shown in Fig. 7(c) with 200 mM ectoine. However, when high concentration (300 mM, 500 mM) of ectoine is added to the solution, as shown in Fig. 7(d) and (e), the condensed DNA structures become looser again. The changing process of DNA morphology is consistent with the result of EM and size of DNA mentioned in last section.

### The condensing force of DNA by magnetic tweezers (MT)

**Fig. 8:**
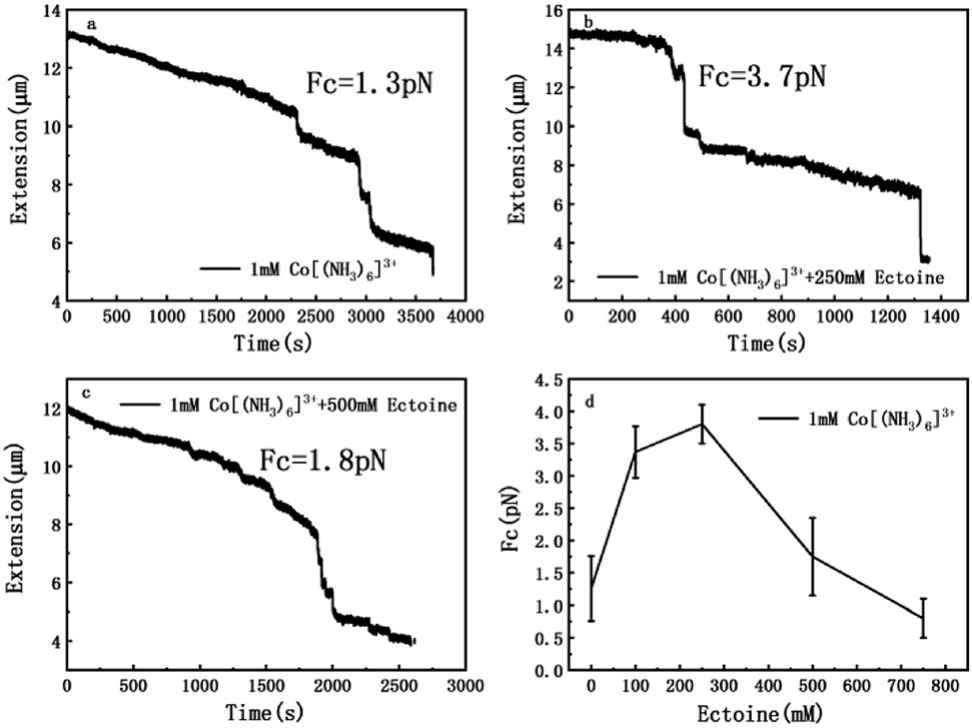
The curve of condensing forces. (a) DNA extension–time curve measured by MT in DNA compaction process with 1 mM [Co(NH_3_)_6_]^3+^. (b) DNA extension–time curve measured by MT in DNA compaction process with 1 mM [Co(NH_3_)_6_]^3+^ + 250 mM ectoine. (c) DNA extension–time curve measured by MT in DNA compaction process with 1 mM [Co(NH_3_)_6_]^3+^ + 500 mM ectoine. (d) *F*_c_ in 1 mM [Co(NH_3_)_6_]^3+^ with different concentration of ectoine. The error bars represent the corrected sample standard deviation.

The measurement of condensing force of DNA by MT can be briefly described as follows: at first, we must find a single tethered λ-DNA, whose extension is close to 16 μm under high tension (>10 pN) in PBS buffer. Then, we flow the solution including 1 mM [Co(NH_3_)_6_]^3+^ and different concentration of ectoine (0 mM, 100 mM, 250 mM, 500 mM, 750 mM) into the sample cell and apply magnetic force to the DNA by moving the magnet slowly to the beads tethered DNA. The condensing force (*F*_c_) is the force when the first step-like shrinking occurs when we lower the applied force by moving back the magnet. Fig. 8(a)–(c) shows the typical curves of DNA condensing process in the solution containing 1 mM [Co(NH_3_)_6_]^3+^ and various concentrations of ectoine. *F*_c_ is 1.3 pN in absence of ectoine and goes up to 3.7 pN when 250 mM ectoine was added in solution. However, the force stops increasing further and goes down when the concentration of ectoine is larger than 250 mM. *F*_c_ becomes 1.8 pN in solution containing 1 mM [Co(NH_3_)_6_]^3+^ and 500 mM ectoine. Fig. 8(d) exhibits an increase of *F*_c_ with increasing concentration of ectoine up to a maximum at 250 mM, followed by a gradual decrease in *F*_c_. The varying trend of *F*_c_ is consistent with the variation of electrophoretic mobility shown in Fig. 4. Initially, the condensing force is weak (1.3 pN) due to the strong coulombic repulsion between DNA segments as its mobility is quite negative (−0.5). Then *F*_c_ increases resulting from the high charge neutralization of DNA by counterions as the mobility becomes less negative (−0.3). Finally, the condensing force goes down gradually since ectoine of high concentration pulls the electrophoretic mobility of DNA back in the negative direction.

**Fig. 9:**
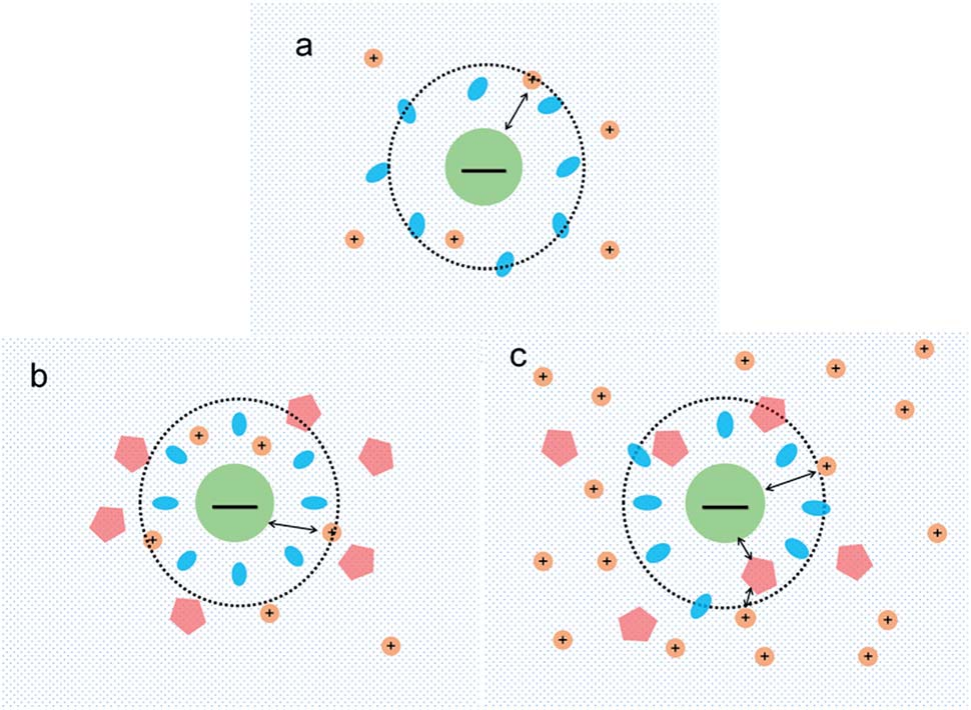
Schematic representation of interactions between DNA and counterions in ectoine solution. The central circle denotes DNA, blue oval as water, small orange circle as cation and pentagon as ectoine (a) interactions between DNA and low concentration of metal ions; (b) interactions between DNA and cations of low concentration in presence of ectoine; (c) interactions between DNA and cations of high concentration in presence of ectoine.

Based on the data on the interaction between ectoine and DNA, we propose a possible molecular mechanism to explain the phenomenon. A broad range of naturally occurring compatible solutes has been proven to protect or stabilize proteins or other bio-molecules.^41^ Although the molecular details of the underlying mechanism are still not fully understood, most of the explanations attribute the resulting protein stabilization mechanism to a preferential exclusion of co-solutes around the macro-molecular compounds.^42–48^ In the numerical simulation of ref. 45, a strong binding to DNA of ectoine was found, and was attributed to the highly negative charge of the DNA phosphodiester backbone *via* strong electrostatic interactions in combination with pronounced dispersion energies. However, this binding is not so effective in the present case since the counterions in at least millimolar concentration exist in solution and they neutralize the most charge of the phosphodiester backbone. Thus, the electrostatic interactions between ectoine and DNA are not as strong as in the condition of the numerical simulation. We believe the preferential exclusion mechanism for ectoine around DNA is still effective in the condition of high ionic strength. In this regard, the co-solute molecules that are repelled from the immediate vicinity of the protein surface are successively replaced by excess water molecules, which stabilize the native form in terms of a preferential hydration mechanism.^49^ In this mechanism, the unfavourable interactions of these substances with the proteins, is a reflection of an increase in the surface free energy of water induced by these additives, hence of the surface tension of water. This must result in the exclusion of the cosolute from the water-macromolecule interface. It was found that ectoine is strongly hydrated, even in the presence of high salt concentrations. In the case of NaCl, sodium ions tend to bind to ectoine, though not very stable.^50^ On the other hand, solutions of the osmolyte ectoine in water exhibit a strong increase of the static relative permittivity with increasing ectoine concentration.^9^ As a consequence of the high permittivity, this osmolyte shields effectively long-range Coulomb interactions among ions in ectoine-containing solutions and hinder the counterion condensation on the surface of DNA. We believe that *via* this effect, which should be common to all zwitterionic osmolytes, ectoine protects against excessive ions within the cell in addition to its strong osmotic activity protecting against ions outside. For charge neutralization of DNA, the two mechanisms have opposite effects. For a typical case of divalent or trivalent counterion in solution, the mobility is promoted when the ionic concentration is in the low range, where the preferential exclusion effect is dominated so that the counterions is much easier to condense on the surface of DNA, implying the charge neutralization is promoted. In the range of high concentration of counterions, the enhancement of dielectric constant of the medium plays an essential role in the interaction between DNA and cations. In this situation, only when ions and/or charged sites come into contact by accident do they “see” each other and possibly aggregate. One may speculate that this effect, exerted by zwitterionic osmolytes, protects biomolecules against excess ions within the cell in addition to their well-known osmotic activity protecting the cell against the adverse effect of excessive salt outside. Therefore, the competitive combination of the exclusion hydration and the enhance of dielectric constant of medium of ectoine provide a reasonable explanation for the simultaneous promotion and suppression of DNA charge neutralization and compaction. This mechanism can be explained schematically in Fig. 9. Fig. 9(a) shows the case of mild counterion concentration without ectoine in solution. In this case, the water shell around DNA is not very organized, and less charge of DNA is neutralized because of low concentration of counterions, implying highly negative electrophoretic mobility and strong Coulombic repulsion. It corresponds to the starting points of the mobility curves in Fig. 2(b) for the case of sodium ions. When ectoine is introduced into the solution, as shown in Fig. 9(b), the water shell becomes more organized and more counterions are attracted to the vicinity of DNA because of the preferential exclusion of the cosolute although the concentration of counterions is the same as in (a). Thus, the charge of DNA is much more neutralized, implying less negative mobility and stronger condensing force of DNA-complex, corresponding to the peaks of the mobility curves in Fig. 2(b). If the concentration of counterions becomes quite high, ectoine can replace water molecule in the hydration shell of DNA, weakening the interaction between DNA and the cations because of the enhancement of relative permittivity, shown in Fig. 9(c), resulting in the descendance of the mobility in Fig. 2(b). Thus, the electrostatic interaction plays a key role in DNA compaction in the mechanism.

## 3. Materials and experimental methods

### Materials

Double strands λ-DNA (48 502 bp) was purchased from New England Biolabs company and the original concentration of DNA was 500 ng μL^−1^. Ectoine (1,4,5,6-tetrahydro-methyl-4-pyrimidinecarboxylic acid, purity ≥ 95.0%), hexaminecobalt(iii) chloride ([Co(NH_3_)_6_]Cl_3_, purity > 99.0%), magnesium chloride hexahydrate (MgCl_2_–6H_2_O, purity > 99.0%), sodium chloride (NaCl, purity > 99.0%), and hydroxylmethyl aminoethane (Tris, purity ≥ 99.8%) were purchased from Sigma-Aldrich (St. Louis, MO, USA), and were used without further purification. Purified water was obtained from a Milli-Q system (Millipore, Billerica, MA, USA). All the buffer used in DLS and AFM were the Tris (10 mM, pH = 7.5). The final DNA concentration in solution is 1 ng μL^−1^.

### Electrophoretic mobility measurement (EM) and size measurement by DLS

The electrophoresis-mobility measurement (EM) were carried out by using a DLS device of Malvern Zetasizer nano ZS90 (Malvern Instruments Limited Company, Malvern, UK) equipped with the patented M3-PALS technique, in which a He–Ne gas laser (*λ* = 633 nm) was used. The light scattering was collected by an avalanche photodiode mounted on the goniometer arm in the perpendicular direction to the incident light. We added DNA to the mixed solution, which including cations (Na^+^, Mg^2+^, [Co(NH_3_)_6_]^3+^) and ectoine ranging from 0–500 mM. All samples measured after 5 min incubation at room temperature. During the measurement, 1 mL volume of DNA solution was used, and the sample cell was kept at 25 °C.

In size measurements, the laser power is automatically attenuated in order to make the count rate from the sample within acceptable limits. Clear disposable capillary cells were used. In sample preparation, we added DNA to the mixed solution, which including cations and ectoine. All samples measured after 10 min incubation at room temperature. During the measurement, 100 μL volume of DNA solution was used, and the sample cell was kept at 25 °C.

### Atomic forced microscopy

The sample preparing procedure can briefly be described as follows: mica (1 × 1 cm^2^) attached to glass slide preparing as substrates for DNA adsorption. We added DNA to the solution, which including 0.01 mM [Co(NH_3_)_6_]^3+^ and ectoine ranged from 0–250 mM. A drop of about 20 μL of solution was deposited for 3 min on a fresh, clear mica surface. The surface was rinsed with distilled water and dried with a gentle flow of nitrogen gas. The prepared samples were scanned by AFM (JPK Nano Wizard III, Berlin, Germany) in AC mode. A 125 μm long and 30 μm wide and 4 μm thickness silicon AFM probe (NCHR-50, Nano World Corporation, Tokyo, Japan) with aluminium coating, spring constant 42 N m^−1^, and resonance frequency of 320 kHz was used. All images were captured from a 5 × 5 μm^2^ viewing area on the sample by a scan rate of 1.0 Hz. Each image was 512 × 512 pixels (4–6 nm per pixel).

### Magnetic tweezers (MT)

**Fig. 10:**
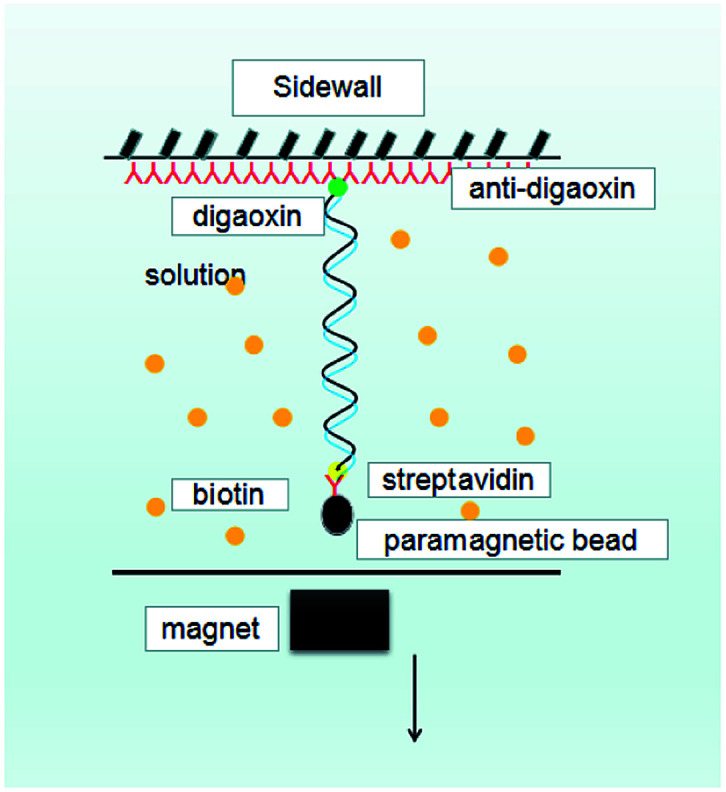
A schematic diagram of MT.

A single molecular magnetic tweezers was used to obtain the force spectroscopy of DNA in counterion solutions. The detail of setup is as described before,^29,51^ as shown in Fig. 10, where the sidewall-DNA-paramagnetic bead structure was presented schematically. The shrinking of a single DNA chain can be monitored by measuring the DNA extension in time, while the tethering force can be altered by moving the magnet back and forth. A video camera was used to monitor the image of the structure in the focal plane, and it was used to record the position of the microsphere in real-time. The analysis of the extension was determined by a tracking algorithm by fast Fourier transform-based correlation techniques.

In the force measurement by the tweezers, we must find a single suspending lambda-DNA, and the bead was pulled to its maximal displacement to the sidewall. Then, the solution containing [Co(NH_3_)_6_]^3+^ (1 mM) with different concentrations of ectoine was flushed into the cell and incubated for 15 minutes. We can move the magnet away slowly to lower the tethering force of DNA. When the force is small enough, a step-like shrinking of DNA occurs and the corresponding condensing force is recorded. When DNA is compacted, the magnetic bead is close to the sidewall to form a compact structure. We can also unravel the DNA condensate by applying a greater force on the bead.

## 4. Conclusions

Based on the present investigation on the interaction between ectoine and DNA, we can draw the following conclusions:

(1) The effect of ectoine on DNA charge neutralization and compaction depends on the valence of counterions in solution. In the case of monovalent counterion, the electrophoretic mobility of DNA increases in the presence of ectoine, implying that the charge neutralization of DNA is promoted when adding extra ectoine. In the cases of divalent and trivalent counterions, however, a mixing effect of promotion and suppression can be found. Specifically, when high concentration of ectoine is present in solution, the mobility is promoted in the range of low concentration of di- or tri-valent cations, while it is suppressed in the range of high counterion concentration.

(2) The promotion and suppression of charge neutralization of DNA corresponds accordingly to the increasing and decreasing of condensing force of DNA, which was measured directly by magnetic tweezers through tethering DNA condensates in solution. Meanwhile, the corresponding change of DNA morphologies has been observed by atomic force microscopy in presence of ectoine.

(3) In a theoretical respect, we proposed a possible mechanism for explaining the experimental phenomenon by combining the preferential exclusion with the enhancement of dielectric constant of medium of ectoine, which is qualitatively consistent to the phenomena observed experimentally.

## Conflicts of interest

The authors declare no conflicts of interest.

## Supplementary Material
